# Ischemic Preconditioning and Postconditioning Protect the Heart by Preserving the Mitochondrial Network

**DOI:** 10.1155/2022/6889278

**Published:** 2022-09-27

**Authors:** Nur Izzah Ismail, Nathaly Anto Michel, Khairunnisa Katwadi, Mim-Mim Lim, To-Kiu Chan, Attaur Rahman, Dachun Xu, Sang-Ging Ong, Derek J. Hausenloy, Sang-Bing Ong

**Affiliations:** ^1^Department of Medicine and Therapeutics, Faculty of Medicine, Chinese University of Hong Kong (CUHK), Hong Kong; ^2^Centre for Cardiovascular Genomics and Medicine (CCGM), Lui Che Woo Institute of Innovative Medicine, CUHK, Hong Kong; ^3^Hong Kong Children's Hospital (HKCH), Hong Kong Hub of Paediatric Excellence (HK HOPE), Kowloon Bay, Hong Kong; ^4^Center for Medical Research, Medical University of Graz, Experimental Cardiology, Stiftingtalstraße 24, 8010 Graz, Austria; ^5^Signature Research Program in Cardiovascular and Metabolic Disorders (CVMD), Duke-NUS Medical School, 8 College Road, Singapore 169857; ^6^Department of Cardiology, Shanghai Tenth People's Hospital, Tongji University School of Medicine, Shanghai 200072, China; ^7^Department of Pharmacology & Regenerative Medicine, The University of Illinois College of Medicine, Chicago, IL, USA; ^8^Division of Pulmonary, Critical Care, Sleep and Allergy, Department of Medicine, The University of Illinois College of Medicine, Chicago, IL, USA; ^9^Yoo Loo Lin School of Medicine, National University of Singapore, Singapore; ^10^National Heart Research Institute Singapore, National Heart Centre Singapore, Singapore; ^11^The Hatter Cardiovascular Institute, University College London, London, UK; ^12^Cardiovascular Research Center, Collage of Medical and Health Sciences, Asia University, Taiwan; ^13^Kunming Institute of Zoology-The Chinese University of Hong Kong (KIZ-CUHK) Joint Laboratory of Bioresources and Molecular Research of Common Diseases, Kunming Institute of Zoology, Chinese Academy of Sciences, Kunming, 650223 Yunnan, China

## Abstract

**Background:**

Mitochondria fuse to form elongated networks which are more tolerable to stress and injury. Ischemic pre- and postconditioning (IPC and IPost, respectively) are established cardioprotective strategies in the preclinical setting. Whether IPC and IPost modulates mitochondrial morphology is unknown. We hypothesize that the protective effects of IPC and IPost may be conferred via preservation of mitochondrial network.

**Methods:**

IPC and IPost were applied to the H9c2 rat myoblast cells, isolated adult primary murine cardiomyocytes, and the Langendorff-isolated perfused rat hearts. The effects of IPC and IPost on cardiac cell death following ischemia-reperfusion injury (IRI), mitochondrial morphology, and gene expression of mitochondrial-shaping proteins were investigated.

**Results:**

IPC and IPost successfully reduced cardiac cell death and myocardial infarct size. IPC and IPost maintained the mitochondrial network in both H9c2 and isolated adult primary murine cardiomyocytes. 2D-length measurement of the 3 mitochondrial subpopulations showed that IPC and IPost significantly increased the length of interfibrillar mitochondria (IFM). Gene expression of the pro-fusion protein, Mfn1, was significantly increased by IPC, while the pro-fission protein, Drp1, was significantly reduced by IPost in the H9c2 cells. In the primary cardiomyocytes, gene expression of both Mfn1 and Mfn2 were significantly upregulated by IPC and IPost, while Drp1 was significantly downregulated by IPost. In the Langendorff-isolated perfused heart, gene expression of Drp1 was significantly downregulated by both IPC and IPost.

**Conclusion:**

IPC and IPost-mediated upregulation of pro-fusion proteins (Mfn1 and Mfn2) and downregulation of pro-fission (Drp1) promote maintenance of the interconnected mitochondrial network, ultimately conferring cardioprotection against IRI.

## 1. Introduction

Myocardial ischemia or occlusion of the coronary artery providing blood flow to the heart is characterized by severe hypoxia, acidosis, energy depletion, and ion homeostasis alterations, leading to cardiac dysfunction and ultimately cell death [1]. The most effective therapeutic option for reducing ischemic myocardial injury and infarct size is prompt and efficient myocardial reperfusion or reoxygenation (re-introduction of blood flow to the heart), but reperfusion itself may lead to an exacerbation of myocardial cell death, due to the sudden burst of oxygen experienced by the cells. This phenomenon is termed acute myocardial ischemia reperfusion injury (IRI) [2, 3], and circumventing IRI remains a major impetus of cardiovascular research to reduce myocardial cell death following infarction and improve outcomes in patients with acute myocardial infarction.

One such approach to prevent IRI lies in targeting the mitochondria. Mitochondria are abundant in the cardiac cells and crucial in determining cardiac cell fate. They function as energy producers and regulators of apoptosis and necrotic cell death. Cardiomyocytes consume energy to “function,” and to accommodate this requirement, these cells host a high density of mitochondria [4]. Studies for the past decade have revealed that mitochondria are dynamic in shape and are highly motile. Mitochondria constantly undergo “fission” to generate discrete fragmented mitochondria or “fusion” to form an interconnected elongated phenotype. Under normal physiological conditions, mitochondria exist in a dynamic equilibrium of elongated and fragmented state [5]. However, the presence of stress skews this balance, and multiple lines of evidence have shown that mitochondrial functional impairments are critical determinants for cardiomyocytes loss during IRI [2, 6, 7]. It has been shown that IRI leads to mitochondrial fission causing the mitochondria to fragment, while reversing this phenotype via genetic or pharmacological means rescues the cardiac cells from IRI-induced cell death [7]. Therefore, an imbalance in the shapes of mitochondrial is considered one of the major mechanisms in the pathogenesis of IRI, and manipulating mitochondrial shapes is therefore an untapped potential new therapy to combat IRI.

Ischemic conditioning encompassing preconditioning (stuttered episodes of ischemia and reperfusion before the index ischemic period) and postconditioning (stuttered episodes of ischemia and reperfusion at the onset of reperfusion) are cardioprotective interventions that protect the heart from IRI in the preclinical setting [8–11]. Many of the signaling pathways conveying the cardioprotective signal of both IPC and IPost appear to converge on the mitochondria [12–16]. Whether IPC and IPost modulates mitochondrial morphology remains unknown. In this study, we aim to determine whether IPC and IPost protect the heart against IRI by modulating cardiac mitochondrial morphology.

## 2. Methods

### 2.1. H9c2 Cell Culture

H9c2 embryonic rat heart-derived (ventricular) cells (myoblast) were purchased from ATCC and cultured in ATTC-formulated Dulbecco's Modified Eagle's Medium (DMEM) supplemented with 10% fetal bovine serum (FBS) in 5% CO_2_ incubator at 37 °C. The cells were subcultured when they reached 70% confluence.

### 2.2. Primary Cardiomyocyte Isolation and Culture

The experimental protocol was approved by the Animal Experimentation Ethics Committee (AEEC), Faculty of Medicine, The Chinese University of Hong Kong (Ref No. 20-147-MIS). Male mice, aged between 8 and 12 weeks old from the PhAM^excised^ (photo-activatable mitochondria) Dendra strain (Jackson Labs, USA) were used in this study. The anesthetization was performed via intraperitoneal (i.p.) injection of ketamine/xylazine (87.5 mg/kg ketamine and 12.5 g/kg xylazine). When no response was observed upon tail or toe pinch, the mouse was then placed in the supine position by gently fixing the forepaws and hindpaws to a pinnable work surface on a table bench. Then, the surgical procedure was performed by dissecting the chest cavity to expose the heart and followed by the heart removal procedure. The cells were isolated by using a simplified Langendorff-free method [17] by enzymatic digestion. The cells were cultured on the laminin-coated glass bottom dishes in 5% CO_2_ incubator at 37 °C.

### 2.3. Preparation of Ischemic and Normoxic Buffer

The basic constituents of normoxic and ischemic buffers were prepared by dissolving 1 mM KH_2_PO_4_, 10 mM NaHCO_3_, 1.2 mM MgCl_2_.6H_2_O, and 25 mM Na-HEPES in of distilled water. For normoxic buffer, NaCl (98 mM), KCl (3 mM), D-glucose (10 mM), Na-pyruvate (2 mM), and 1.26 mM CaCl_2_ were added to the basic constituent, and the solution was bubbled with carbogen gas; pH was adjusted to 7.4. The ischemic buffer was prepared by dissolving NaCl (74 mM), 0.55 g KCl (16 mM), Na-Lactate (20 mM), and CaCl_2_ (1.26 mM) into the basic constituent, and the solution was aerated with nitrogen to remove the oxygen in the buffer; pH was adjusted to 6.2. Both normoxic and ischemic buffers were filtered through a 0.2 *μ*m filter, and 1% of antibiotic (Pen/Strep) was added to the buffer [7].

### 2.4. Preparation of Krebs-Henseleit Buffer (KHB)

The KHB was prepared by adding 118.5 mM NaCl, 25.0 mM NaHCO_3_, 4.7 mM KCl, 1.2 mM MgSO_4_, 1.2 mM KH_2_PO_4_, 11.0 mM glucose, and 2.5 mM CaCl_2_, and pH was set to 7.4 at 37 °C. The buffer was freshly prepared and aerated with carbogen gas that consists of 5% CO_2_ and 95% O_2_ prior to the experiment.

### 2.5. In Vitro Simulated Ischemia Reperfusion Injury Model in H9c2 Cells and Primary Adult Cardiomyocytes

An IRI model was simulated in both H9c2 cells and the isolated primary adult cardiomyocytes. Both cells were exposed to different experimental condition such as ischemic and reoxygenation times to evaluate the optimized ischemic and reoxygenation time for both cells' model. The H9c2 cells were subjected to 3 hours of simulated ischemia followed by various durations of reoxygenation (1.5, 4, and 24 hours). For adult primary cardiomyocytes, the cells were subjected to 30 and 45 minutes of simulated ischemia followed by 30 minutes of reoxygenation [7]. To simulate ischemic conditions, cells were cultured in ischemic buffer in a controlled hypoxic plastic chamber. To remove the oxygen in the chamber, nitrogen gas was flushed into the chamber for 5 min with a pressure lower than 2 psi. The chamber was then incubated in 5% CO_2_ incubator with temperature at 37 °C with the incubation time depending on the protocol for ischemia. Reoxygenation was conducted by replacing the ischemic buffer with normoxic buffer, and cells were incubated in normoxic incubator at 37 °C. For time-controlled samples, the cells were left in normoxic buffer in 5% CO_2_ incubator during the length of the experiments.

### 2.6. Langendorff-Isolated Heart Model

The heart was mounted on the Langendorff perfusion apparatus and the aorta perfused with oxygenated Krebs-Henseleit buffer (KHB) at a constant flow of 4 ml/min (ᵙ 7.6 rpm), and temperature was kept at 37 °C throughout the procedure. The heart was equilibrated and stabilized by allowing the KHB flow through the system for 10 min before the initiation of the experiment. The mice hearts were subjected to 20 minutes of global ischemia, no-flow, normothermic ischemia (37 °C) by clamping the perfusate line and then followed by 60 minutes of reperfusion by unclamping the perfusate line. During ischemia, in order to regulate the experimental temperature, the heart was immersed in perfusion buffer KHB at 37 °C [18].

### 2.7. IPC and IPost

Different algorithms of IPC and IPost were induced in the different models of H9c2, isolated adult primary cardiomyocytes, and the Langendorff isolated perfused heart. In the H9c2 cells, IPC was induced by 30 minutes of simulated ischemia (sI) followed by 30 minutes of reoxygenation, prior to the index ischemic period of 3 hours and a subsequent 4 hours of reoxygenation. The IPost algorithm consists of 3 cycles of 10 minutes of sI and 10 minutes of reoxygenation, at the onset of reoxygenation [19].

In the murine cardiomyocytes, IPC was achieved by 10 minutes of sI followed by 10 minutes of reoxygenation, prior to the index ischemic period of 30 minutes of sI followed by 30 minutes of reoxygenation. The IPost algorithm consists of 10 minutes sI and 10 minutes of reoxygenation, at the onset of reoxygenation [20].

In the Langendorff isolated heart perfusion model, IPC was achieved by 3 cycles of 10 minutes of global ischemia followed by 10 minutes of reperfusion, prior to the index of ischemia for 20 minutes and a subsequent 60 minutes of reperfusion. The IPost was achieved by 3 cycles of 10 minutes of global ischemia and 10 minutes of reperfusion, at the onset of the reperfusion [21].

### 2.8. Assessment of Cardiac Cell Death

Cell death of both H9c2 and cardiomyocytes was assessed by propidium iodide (PI) staining using the fluorescent microscope as previously described [7, 20]. Four images were randomly captured for each experiment setup (*n* = 4 independent experiments) [7].

### 2.9. Assessment of Myocardial Infarct Size

Infarct size of the whole heart was determined using triphenyltetrazolium chloride (TTC) staining. The heart sections were incubated in 1% TTC in PBS, at 37 °C for 10 minutes. Subsequently, the heart slices were fixed in 10% natural buffered formalin solution. The heart slices were photographed using a light microscope, and the measurement of the infarct size was done by using the “colour threshold” mode in the ImageJ software to accurately quantify the percentage of infarct size as a proportion of the total heart volume [22].

### 2.10. Mitochondrial Morphology in H9c2 Cells and Primary Cardiomyocytes

**Figure 1:**
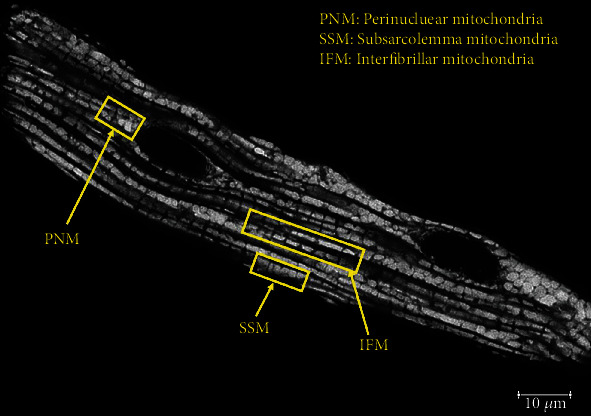
Subpopulations of mitochondria in the isolated primary adult cardiomyocyte. Representative 2D confocal image of the 3 mitochondrial subpopulations in the isolated primary adult cardiomyocyte: interfibrillar (IFM), subsarcolemmal (SSM), and perinuclear (PNM) mitochondria, observed with 100X oil immersion lens.

H9c2 cells and primary cardiomyocytes were fixed with 4% paraformaldehyde (PFA), and H9c2 cells were stained with MitoView Green. Mitochondrial morphology was determined using a Leica TCS SP5 confocal microscope equipped with a 100 X oil immersion objective. For the H9c2 cells study, 80 cells per group (sIR, IPC, and IPost) were randomly selected and imaged (*n* = 4 independent experiments with 20 cells per experiment). The cells were classified as containing either predominantly (>50%) elongated or predominantly (>50%) fragmented mitochondria [7] based on visual observation. For the primary cardiomyocytes, 10 cells per group (sIR, IPC, and IPost) were randomly selected for imaging.  Figure 1 shows representative image of mitochondrial morphology in a single cell of primary adult cardiomyocytes. The length of the mitochondrial in the different subpopulations—perinuclear (PNM), interfibrillar (IFM), and subsarcolemmal (SSM)—was measured using ImageJ. A straight line was drawn along the maximal length of a single mitochondrion of on a 2D image. Only mitochondria with clear delineation were chosen and measured. Ten cells per group (sIR, IPC, and IPost) were randomly selected for imaging, and each experiment was repeated 4 times.

### 2.11. Quantification of mRNA Levels of Mitochondria-Shaping Proteins by qPCR

Total RNA was isolated using RNeasy mini kit according to the manufacturer's instructions. Complementary DNA (cDNA) was synthesized by using iScript cDNA synthesis kit according to the protocol provided by the manufacturer. The mRNA expression level of mitochondrial-shaping proteins (Mfn1, Mfn2, Opa1, and Drp1) was determined by qPCR using Fast SYBR Green master mix. The GAPDH was used as the internal control; the primers used are listed in Tables 1 and 2. All primers were purchased from Integrated DNA Technology.

### 2.12. Statistical Analysis

The data presented were expressed as mean ± standard error of mean (SEM), unless otherwise stated. Statistical analysis was assessed by Student *t*-test or one-way ANOVA followed by Tukey's post hoc test where appropriate. All statistics were calculated by Prism GraphPad 5.0 (GraphPad Software Inc., San Diego, CA, USA). An error probability of *P* < 0.05 was defined as statistically significant.

## 3. Results

### 3.1. IPC and IPost Protect against IRI

**Figure 2:**
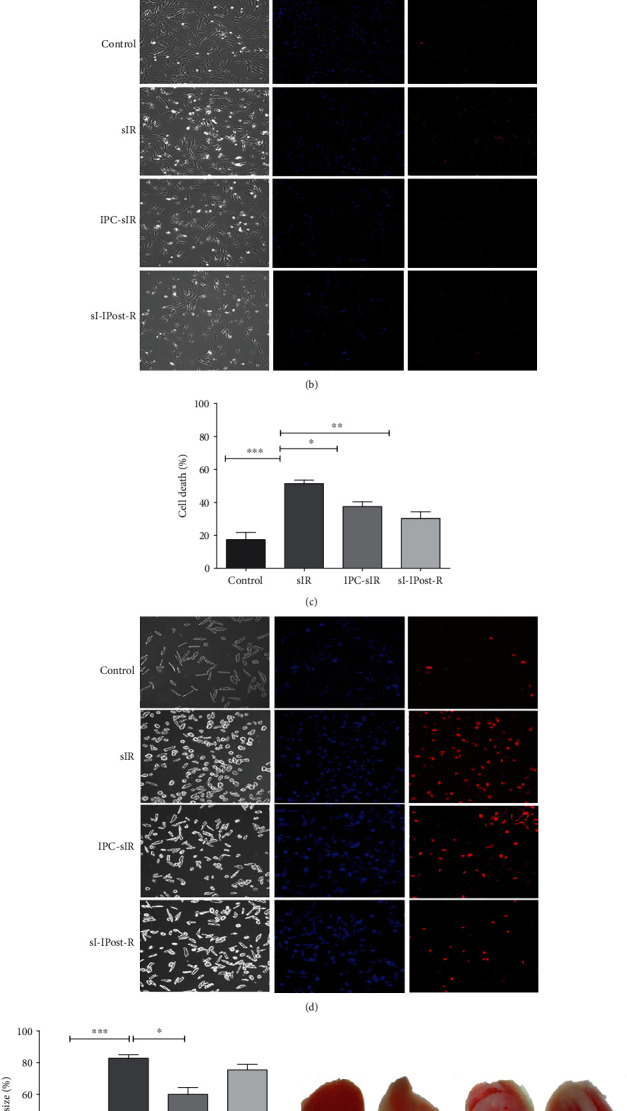
IPC and IPost protects against IRI. (a) Cell death in the H9c2 cardiac cell line following sIR, IPC-sIR, and sI-IPost-R (*n* = 4, ~100 cells per group), ^∗∗^*P* < 0.01 and ^∗∗∗^*P* < 0.005. (b) Representative bright-field and fluorescent images of PI (red panel) and Hoechst (blue panel) staining of H9c2. (c) Cell death in isolated primary adult cardiomyocytes following sIR, IPC-sIR, and sI-IPost-R, (*n* = 4, ~70 cells/field); ^∗^*P* < 0.05, ^∗∗^*P* < 0.01, and ^∗∗∗^*P* < 0.005. (d) Representative bright-field and fluorescent images of PI (red panel) and Hoechst (blue panel) staining of isolated primary adult cardiomyocytes. (e) Myocardial infarct size following IR, IPC-IR, and I-IPost-R, *n* = 3 hearts per group; ^∗^*P* < 0.05 and ^∗∗∗^*P* < 0.005. (f) Representative image of heart slices of control, post-IR, IPC-R, and I-IPost-R.

In H9c2 cells, IPC and IPost significantly reduced the percentage of cell death to 30.7 ± 2.7% and 32.5 ± 3.8%, respectively, compared to sIR which was 45.5 ± 1.4%, *n* = 4, *P* < 0.01 (Figures 2(a) and 2(b)). In primary cardiomyocytes, cell death was significantly reduced in both IPC and IPost group compared to the sIR group (IPC: 37.4 ± 3.01%, *n* = 4, and *P* < 0.05; IPost: 30.2 ± 4.1%, *n* = 4, and *P* < 0.01 compared to sIR which is 51.4 ± 2.1%) (Figures 2(c) and 2(d)). In the Langendorff mouse model, infarct size was reduced significantly to 60.0 ± 4.3% by IPC and 75.4 ± 3.4% by IPost as compared to the sIR group (82.8 ± 2.2%), *n* = 3, *P* < 0.05 (Figures 2(e) and 2(f)).

### 3.2. IPC and IPost Preserves Mitochondrial Morphology

**Figure 3:**
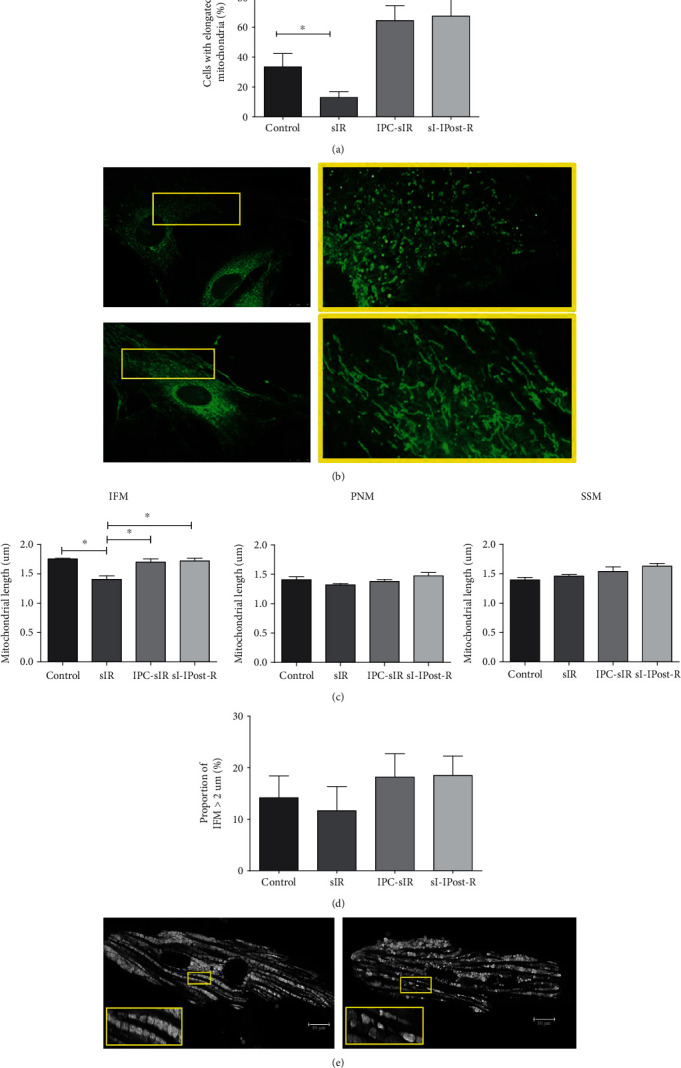
IPC and IPost promotes mitochondrial elongation in cardiac cells. (a) Proportion of cells with elongated mitochondria following sIR, IPC-sIR, and sI-IPost-R (*n* = 4, ~ 70 cells/field); ^∗^*P* < 0.05 and ^∗∗^*P* < 0.01 (b) Representative confocal images of fragmented mitochondria (upper panel) and elongated mitochondria (lower panel) in H9c2 cells, observed using 100X oil immersion lens. (c) Average mitochondrial length in the different subpopulation of mitochondria (~2000 IFM/group, ~1000 PNM/group, ~1000 SSM/group; ^∗^*P* < 0.05). (d) Proportion of IFM with length of >2 *μ*m in isolated primary adult primary cardiomyocytes (*n* = 4 mice; 60 cells/group; ~2000 IFM/group, ~1000 PNM/group, and ~1000 SSM/group). (e) Representative confocal images of mitochondrial morphology in isolated adult primary cardiomyocytes (left panel: elongated mitochondria; right panel: fragmented mitochondria).

Following sIR, the proportion of H9c2 cells with predominantly elongated mitochondria reduced significantly to 13.0 ± 3.9% compared to the control which was 33.5 ± 8.9% (*n* = 4 and *P* < 0.05). Conversely, IPC and IPost treatment significantly increased the percentage of cells with elongated mitochondria to 64.5 ± 9.9% (*n* = 4 and *P* < 0.005) and 67.5 ± 13.7% (*n* = 4 and *P* < 0.01, respectively) as compared to the sIR group (Figure 3(a)).  Figure 3(b) shows the representative images of elongated and fragmented mitochondria in H9c2 cells.

Assessment of maximal mitochondrial length in primary cardiomyocytes demonstrated that sIR significantly affects the length of IFM, compared to the control group (Figure 3(c)). The average length of the IFM was 1.41 ± 0.06 *μ*m in sIR versus 1.76 ± 0.01 *μ*m in control. IPC and IPost significantly elongated the average length of IFM (1.70 ± 0.05 *μ*m and 1.72 ± 0.04 *μ*m, respectively) compared to sIR group (2000 = IFM/group, *P* < 0.05). The average length of the PNM was 1.35 ± 0.02 *μ*m in sIR versus 1.41 ± 0.05 *μ*m in control group. The average of PNM in IPC and IPost group was 1.38 ± 0.03 *μ*m and 1.47 ± 0.06 *μ*m, respectively (*n* = 1000 PNM/group, *P* > 0.05; N.S). The average length of SSM was 1.46 ± 0.02 *μ*m in sIR compared to 1.39 ± 0.04 *μ*m in control group. The average of SSM in IPC and IPost group was 1.54 ± 0.07 *μ*m and 1.63 ± 0.04 *μ*m, respectively (*n* = 1000 SSM/group, *P* > 0.05; N.S).

To further assess the extent of elongation in the IFM mitochondria, the proportion of IFM with length equivalent to a sarcomere or more (> 2 *μ*m) was determined (Figure 3(d)). The proportion of elongated mitochondria in sIR was 11.64 ± 4.69% compared to control (14.16 ± 4.22%). In the presence of IPC and IPost, the proportion of elongated mitochondria was increased to 18.17% ± 4.53 and 18.49 ± 3.73%, respectively, compared to the sIR group, albeit not significant (*n* = 2000 IFM/group, *P* > 0.05; N.S).  Figure 3(e) shows the representative images of mitochondrial morphology in primary cardiomyocytes.

### 3.3. IPC and IPost Modulate Different Mitochondrial-Shaping Proteins

To study the mRNA expression profile of mitochondria-shaping proteins following sIR, IPC, and IPost, mRNA fold changes for Mfn1, Mfn2, Opa1, and Drp1 were quantified using qPCR.

**Figure 4:**
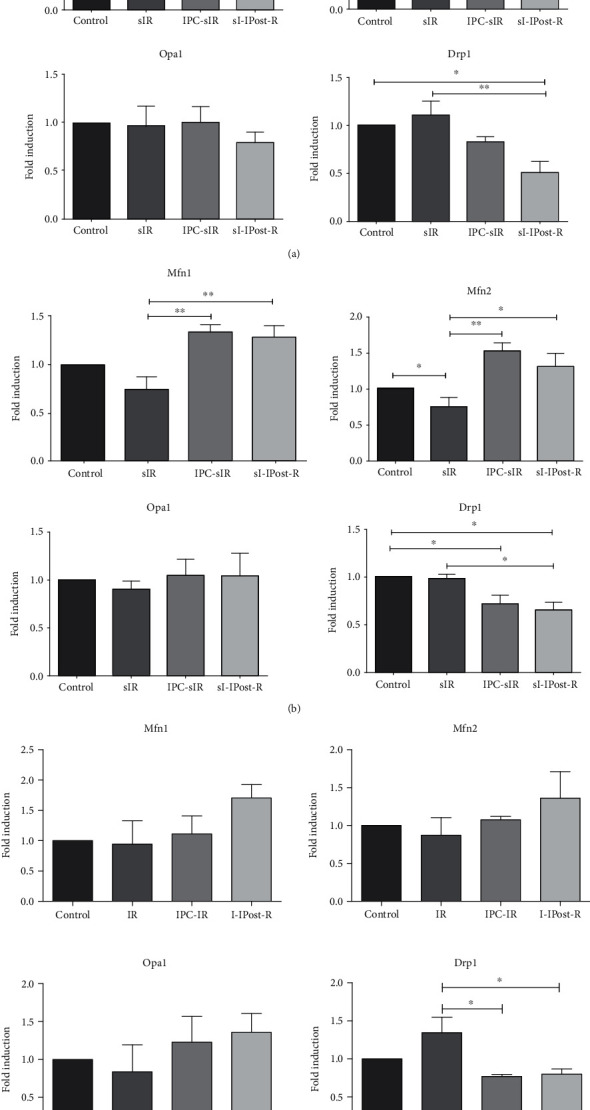
IPC and IPost modulate different mitochondrial-shaping proteins to promote mitochondrial elongation. mRNA levels of different mitochondrial-shaping proteins normalized to GAPDH in (a) H9c2 cardiac cell line following sIR, IPC-sIR, and sI-IPost-R. (b) Isolated primary adult cardiomyocytes. (c) Langendorff-isolated perfused hearts. *N* = 4, ^∗^*P* < 0.05, and ^∗∗^*P* < 0.01.

In the H9c2 cell, sIR reduced the mRNA expression of Mfn1 significantly by 0.6 ± 0.11-fold compared to control but upregulated by IPC (1.59 ± 0.03-fold) compared to control and sIR (*n* = 4 and *P* < 0.05). While sIR also significantly reduced the expression level of Mfn2, no changes in Mfn2 expression were detected in the presence of IPC and IPost. The mRNA level of Drp1 was slightly upregulated by sIR (1.09 ± 0.02-fold), but reduced significantly following IPost (0.5 ± 0.01-fold), when compared to control and sIR (*n* = 4 and *P* < 0.01) (Figure 4(a)).

In the terminally differentiated isolated adult cardiomyocytes, the mRNA expression level of Mfn1 was downregulated by0.7 ± 0.14 fold following sIR as compared to control but upregulated by IPC (1.34 ± 0.07-fold; *n* = 4, *P* < 0.01) and IPost (1.28 ± 0.12-fold; *n* = 4, *P* < 0.01) as compared to sIR. sIR also significantly decreased Mfn2 by 0.74 ± 0.14-fold (*n* = 4 and *P* < 0.05) as compared to control, while IPC and IPost significantly upregulated Mfn2 by 1.51 ± 0.12-fold (*n* = 4 and *P* < 0.01) and 1.31 ± 0.17-fold (*n* = 4 and *P* < 0.05), respectively, as compared to sIR. Although not significant, Opa1 was downregulated by sIR (0.89 ± 0.10-fold), but upregulated by IPC and IPost by 1.04 ± 0.18-fold-and 1.05 ± 0.23-fold, respectively; the expression level of Drp1 was significantly downregulated by IPost (0.66 ± 0.08-fold; *n* = 4, *P* < 0.05) compared to control (Figure 4(b)).

In the Langendorff-isolated perfused heart, the mRNA expression level of Mfn1 was downregulated by sIR (0.94 ± 0.38-fold) as compared to control but upregulated by IPC (1.11 ± 0.29-fold) and IPost (1.70 ± 0.22-fold) compared to sIR. The mRNA expression level of Mfn2 was downregulated by sIR (0.87 ± 0.23-fold) and upregulated by both IPC and IPost (1.08 ± 0.04-fold and 1.36 ± 0.35-fold, respectively). The mRNA expression level of Opa1 was downregulated by sIR by 0.84 ± 0.36-fold compared to control and upregulated by IPC and IPost (1.23 ± 0.34-fold and 1.36 ± 0.25-fold, respectively). For pro-fission protein, Drp1, the mRNA expression level was significantly upregulated by sIR 1.34 ± 0.20-fold as compared to control, while IPC and IPost significantly downregulated the Drp1 level by 0.77 ± 0.03-fold and 0.80 ± 0.06-fold, respectively (*n* = 3 and *P* < 0.05) (Figure 4(c)).

## 4. Discussion

The main findings in this study include: (1) IPC and IPost mediate an increase in proportion of cells with elongated mitochondria following IRI; (2) the numbers of elongated IFM mitochondria were significantly higher following IPC and IPost but not in SSM and PNM; and (3) the profile of mitochondrial-shaping proteins varied across species and cardioprotective intervention administered.

Although IPC/IPost has been extensively studied to facilitate a successful translation to the bedside, the results so far have been inconclusive. In the laboratory, IPC/IPost has been demonstrated to reduce myocardial infarct size in various animal models of AMI [23, 24]. The cardioprotective benefits, however, have not been translated into the clinical setting [25–27]. In large double-blinded, multicenter trials of patients undergoing cardiovascular surgery, remote IPC applied remotely failed to improve clinical outcomes in both short term (RIPHeart) [28] as well as long term (ERICCA) [29]. Similarly, IPost in large-scale trials of patients with ST-segment-elevation myocardial infarction undergoing primary PCI failed to improve myocardial reperfusion and myocardial salvage index nor reduce infarct size in the short term [30, 31] as well as long term [32]. Various factors have been postulated to undermine the failure to translate from bench to bedside, such as the selection of pre- and perioperative anesthesia used, presence of comorbidities, and concomitant medications [33]. In light of these postulations, the role of the mitochondria in ensuring the success of IPC/IPost appeared to have “escaped the radar” of the investigations. In particular, the role of cardiac mitochondrial dynamics in the settings of IPC/IPost remains elusive. We and others have previously demonstrated that tilting the balance of mitochondrial dynamics confers protection against cardiac IRI by desensitizing the opening of the mitochondrial permeability transition pore (mPTP) [7, 20, 34–38] The elongated mitochondria have been purported to sustain increased calcium overload, ROS production, and defective mtDNA, thus conferring cardioprotection [39–43]. The fragmented mitochondria have also been speculated to protect via facilitating clearance of damaged mitochondria and dispersing effects of calcium overloading [36, 44–48]. Although the cardioprotective benefits of modulation of mitochondrial morphology in the settings of cardiac IRI has been demonstrated, this is the first study, to our knowledge, that directly demonstrates the effect of IPC/IPost in preserving cardiac mitochondrial network.

Among the 3 subpopulations of mitochondria in the cardiomyocytes, the effect on IPC/IPost-mediated elongation of mitochondria was only observed in the IFM, with no changes to the SSM and PNM. We have previously put forth the notion that elongated IFM be normalized against the length of an individual sarcomere at approximately 2 *μ*m [7, 20]. The notion is further corroborated by the findings in this current study whereby a similar pattern of increased proportion of IFM with length of >2 *μ*m was observed following IPC/IPost. Although the deleterious effects of IRI have been previously attributed to damage to the SSM [49–52], the technical aspects of the previous studies, e.g., mitochondrial isolation for length measurements and timing of morphology assessment, may partially explain why the SSM was implicated. In the current study, mitochondrial morphology assessment was performed on all clear, high-resolution, and properly defined mitochondria within the cell using Fiji software on 2D confocal images obtained post-reperfusion, whereas other studies assessed the mitochondria post-ischemia. While SSM may indeed, be crucial for the function of mitochondrial Cx43 in mediating cardioprotective benefits of IPC [53], IFM is indispensable for buffering of cytosolic calcium, optimal homeostasis of cardiac energetics, and generation of antioxidant via an association with SR [52, 54, 55]. PNM, conversely, has been associated with provision of energy for transcription [56, 57].

Interestingly, our findings demonstrated that IPC and IPost, although converging on increasing the proportion of cells with elongated mitochondria, seem to mediate this through modulation of different mitochondrial-shaping proteins. As both IPC and IPost have been demonstrated to activate the PI3K/Akt pathway, the elongation of mitochondria observed following IPC/IPost may be mediated by the upregulated Akt [20] Although the activation of Akt has been previously associated with downstream Mfn1-mediated mitochondrial elongation, we found that IPC/IPost mediate different mitochondrial-shaping proteins. IPC mediates an upregulation of Mfn1 which would align with the induction of Akt [20], while a downregulation of Drp1 is crucial for reducing IR-mediated mitochondrial fission and mPTP opening [7]. Surprisingly, while Mfn2 is known for pleiotropic effects in mediating both mitochondrial fusion and tethering of the mitochondria to the SR [58–60], the change in Mfn2 was only detected in the isolated adult cardiomyocytes. A possible rationale for this may be that Mfn2 is differentially expressed in the cardiac cells consisting of fibroblasts, endothelial cells, and cardiomyocytes, thus compensating for the increase observed in the cardiomyocytes. Opa1 has been postulated to play a role in mediating proper mitochondrial respiration whereby it maintains the assembly and formation of respiratory supercomplexes [61, 62]. However, we failed to observe any changes in Opa1 in the current study. To explain this, we reason that the function of Opa1 in mediating IMM mitochondrial fusion is probably secondary to fusion of the OMM by the mitofusins. In addition, the maintenance of the respiratory supercomplexes may be compensated by the presence of other proteins [63–66], thus obviating the need for changes to Opa1 profile. Another crucial factor to take into account is the fact that the importance of Opa1 in protecting against death may only be realized during its post-translational modification into different isoforms [67–70], as opposed to the transcriptional level observed in this study. Drp1 reduction as observed in the H9c2 cells, isolated primary cardiomyocytes, and ex vivo Langendorff hearts is mirrored by the decrease in fragmented mitochondria post-IPC/IPost and served to reinforce the notion that fragmented mitochondria in the settings of acute IR is detrimental probably via sensitization to mPTP opening and release of cytochrome *c*. Whether IPC/IPost modulates the upstream calcium/calcineurin pathway, posttranslational modification of Drp1 or the Drp1-docking proteins Mff or Fis1 [71] remain to be investigated. One point to note, however, is that the mRNA levels of these mitochondrial-shaping proteins were detected post-reperfusion. It would be interesting to investigate the profile of these proteins immediately post-IPC versus post-IPost.

## 5. Conclusion

In summary, the IPC/IPost-mediated mitochondrial elongation may serve to be a modulating target to enhance the efficacy of these cardioprotective strategies as well as a plausible target for successful translation of these strategies to the bedside. Our study put forth the notion that the state of the mitochondria morphology pre- and post-intervention may exert a role in conferring cardioprotection as an endpoint. In this regard, if the mitochondria are fragmented excessively during injury, will interventions restore the mitochondrial morphology adequately to achieve protection? Future trials may consider assessing the cardiac mitochondrial morphology prior to administering interventions as well as post-intervention for enhanced prognostics. The study may benefit from further investigations elucidating how exactly does the elongated mitochondria protect following IPC/IPost, the protein levels of the different mitochondrial-shaping proteins, and the difference in post-translational modifications of the mitochondrial-shaping proteins and whether pharmacological modulation of mitochondrial morphology will enhance or disrupt the cardioprotection conferred by IPC/IPost.

## Figures and Tables

**Table 1:** Mitochondrial-shaping proteins primers for H9c2 cells.

Gene(s)	Primer's sequence (5′– 3′)	Melting temperature (°C)	Product size (bp)
OPA1	Forward: CAGCTGGCAGAAGATCTCAAG Reverse: CATGAGCAGGATTTTGACACC	58.72 57.50	107
Mfn1	Forward: GGAGATACAGGGCTACAGAAAC Reverse: AGCTCTTGCCACTACTTGTC	57.94 57.54	104
Mfn2	Forward: TTGACTCCAGCCATGTCCAT Reverse: GGTGACGATGGAGTTGCATC	59.01 58.99	100
Drp1	Forward: TGGAGATGGTGGTCAGGAAC Reverse: TTTCGTGCAACTGGAACTGG	59.02 58.98	174
GAPDH	Forward: TGCCCCCATGTTTGTGATG Reverse: GCTGACAATCTTGAGGGAGTTGT	58.33 60.81	64

**Table 2:** Mitochondrial-shaping proteins primers for primary cardiomyocytes.

Gene(s)	Primer's sequence (5′–3′)	Melting temperature (°C)	Product size (bp)
OPA1	Forward: ACCTTGCCAGTTTAGCTCCC Reverse: TTGGGACCTGCAGTGAAGAA	59.96 59.16	82 bp
Mfn1	Forward: GATCCGATTCCGAGCTTCCG Reverse: GATCCGATTCCGAGCTTCCG	59.75 60.66	83 bp
Mfn2	Forward: TGCACCGCCATATAGAGGAAG Reverse: TCTGCAGTGAACTGGCAATG	59.66 58.76	78 bp
Drp1	Forward: ATGCCAGCAAGTCCACAGAA Reverse: TGTTCTCGGGCAGACAGTTT	59.89 59.53	86 bp
GAPDH	Forward: AGGTCGGTGTGAACGGATTTG Reverse: TGTAGACCATGTAGTTGAGGT	60.88 55.73	123 bp

## Data Availability

All data are contained within the article.
